# Biofilms and disinfection by-products in Mediterranean drinking water networks: effect of temperature on disinfection strategies

**DOI:** 10.1007/s11274-026-05101-x

**Published:** 2026-08-03

**Authors:** Isabel Volz Gonzalez, Andross Pérez Lleó, Pura Almenar Llorens, María Pedro Monzonís, Isabel Douterelo

**Affiliations:** 1 School of Mechanical, Aerospace and Civil Engineering, The University of Sheffield, Sheffield, UK; 2 Global Omnium Group, Empresa Mixta Valenciana de Aguas SA – EMIVASA, Valencia, Spain

**Keywords:** Biofilm, Chlorine, Drinking water distribution systems, Disinfection by-products, Metals

## Abstract

Biofilms inhabiting drinking water networks can degrade water quality, leading to disinfectant depletion, pipe corrosion and organoleptic issues. In this study, the use of *in situ* biofilm sampling devices, which enabled sample collection without interrupting the supply, provided insights into physicochemical and biological interactions within a network in Valencia (Spain). The devices were installed in an operational chlorinated network and monitored over a year to assess biofilm dynamics. Water quality was evaluated using standard methods for faecal indicator bacteria (FIB) and pathogens, alongside physicochemical parameters including disinfection by-products (DBPs), geosmin, and metals. Biofilms devices were sampled every 3 months to examine development and biofilm regrowth under seasonal conditions. Bacterial communities were characterised using 16S rRNA gene amplicon sequencing and analysed in relation to environmental factors. No FIB or pathogens were detected in any water sample, yet DNA analysis in biofilms (particularly in 6- and 9-month-old biofilms) showed the presence of sequences related with potential opportunistic pathogens including *Legionella* spp. and *Staphylococcus* spp. DBPs, increased during warmer months coinciding with higher chlorine dosages and iron and manganese concentrations. A core microbiome formed mainly by species of *Pseudomonas* spp. and *Acidovorax* spp. was found in water and biofilm samples. A clear temporal succession in biofilm composition was observed, with diversity peaking at mid-developmental stages, yet regrowth was seasonally independent. The installation of biofilm sampling devices in real networks was effective at monitoring microbial dynamics, providing key information for distribution infrastructure management including optimisation of cleaning and disinfection strategies.

**Supplementary Information:**

The online version contains supplementary material available at 10.1007/s11274-026-05101-x.

## Introduction

Based on World Health Organization guidelines (World Health Organization 2017), national and international water authorities are committed to provide safe access to drinking water. The supply of safe drinking water has been a persistent challenge for water utilities worldwide. Despite significant progress having been made to develop treatment strategies to maintain the quality and safety of water; after leaving the treatment plants, its quality can deteriorate through distribution networks (Mian et al. 2019; Bertelli et al. 2018). Research is still needed to fully understand the complex interactions occurring within these networks, and to update traditional treatment, monitoring and management strategies to guarantee a sustainable drinking water supply.

Drinking water in Mediterranean cities such as Valencia (Spain) is considered safe for consumption. However, biofilm development on internal pipe surfaces remains a challenge for water utilities when it comes to managing buried water infrastructure. Biofilms consist of a diverse range of microorganisms (e.g. bacteria, fungi, viruses, etc.) that are associated within a matrix of extracellular polymeric substances (EPS) produced by them (Wingender and Flemming 2011). Protected by the EPS, microorganisms within biofilms obtain ecological benefits like adhesion and a strong cohesion to surfaces, capacity to degrade a wide range of compounds, and protection against external factors such as disinfectants (Wingender & Flemming, 2010, 2011). Protected in this matrix, biofilms (attached or when mobilised) can generate problems in the network including: (i) increased disinfectant consumption, forcing an increase in the applied doses (Berry et al. 2006; Flemming et al. 2016); (ii) production of disinfectant by-products (DBPs) (Mian et al. 2021); (iii) aesthetic deterioration of the water (i.e. odour, taste and discoloration) (Berry et al. 2006; Moreno et al. 2024); (iv) proliferation of opportunistic pathogens and operational problems due to reduced water speed (Learbuch et al. 2022; Moreno et al. 2024); and (v) impacts on hydraulic performance of the network (Flemming et al. 2016).

The current monitoring approach to determine the quality of drinking water for human consumption relies on testing discrete tap water samples. However, the determination of a range of physicochemical and indicator biological parameters in these water samples is not enough to guarantee good quality and safe water at tap level. The monitoring of Drinking Water Distribution System (DWDS) should include regular biofilm growth checks since changes in environmental conditions can promote the growth of harmful pathogens in biofilms that can then be mobilised into the bulk water (Pullerits et al. 2020; Calero et al., 2022). In Mediterranean countries, elevated temperatures during summer months in the water distribution network (> 20 °C) can promote microbial growth, further influencing the development of biofilms and the proliferation of undesirable microorganisms including pathogenic species (van der Wielen et al. 2023; Del Olmo et al. 2021; Calero et al., 2021). Regular biofilm monitoring in the network can facilitate the implementation of preventive and/or corrective actions before the water is supplied to consumers. However, monitoring of biofilms in buried drinking water networks is difficult and not a common practice among water utilities. Nowadays, there are commercially available sensors that can detect biofilm growth (Davis et al., 2021) yet they do not estimate their microbiological composition and the presence of harmful microbes. These sensors, combined with microbial biofilm analysis, can be the key to successfully maintaining a healthy ecosystem within the pipes and to determine when the network needs to be cleaned, levels of residual disinfectant adjusted or in certain cases pipe sections replaced (Turolla et al. 2019; Kitajima et al. 2021).

In this study, biofilms and their behaviour were monitored using *in situ* biofilm sampling devices installed for the first time in a real network in the city of Valencia. Traditional monitoring strategies are mainly based on bulk water or tap water sampling. These samples are not fully representative of the microbial community attached to the surfaces of the pipes and consequently do not reflect the dynamics of communities within DWDS. The sampling devices used in this study, allow monitoring of biofilms *in situ* without disruption of the boundary hydraulic conditions in the pipes (Douterelo et al. 2016). Through these devices the local water utility was able to monitor microbiological processes in the water supply network without stopping the supply. The results obtained informed the water utility on cost-effective management strategies including better maintenance and cleaning of pipe infrastructure to prevent adverse water quality problems.

## Materials and methods

### Installation of sampling devices and collection of biofilm and water samples

**Fig. 1:**
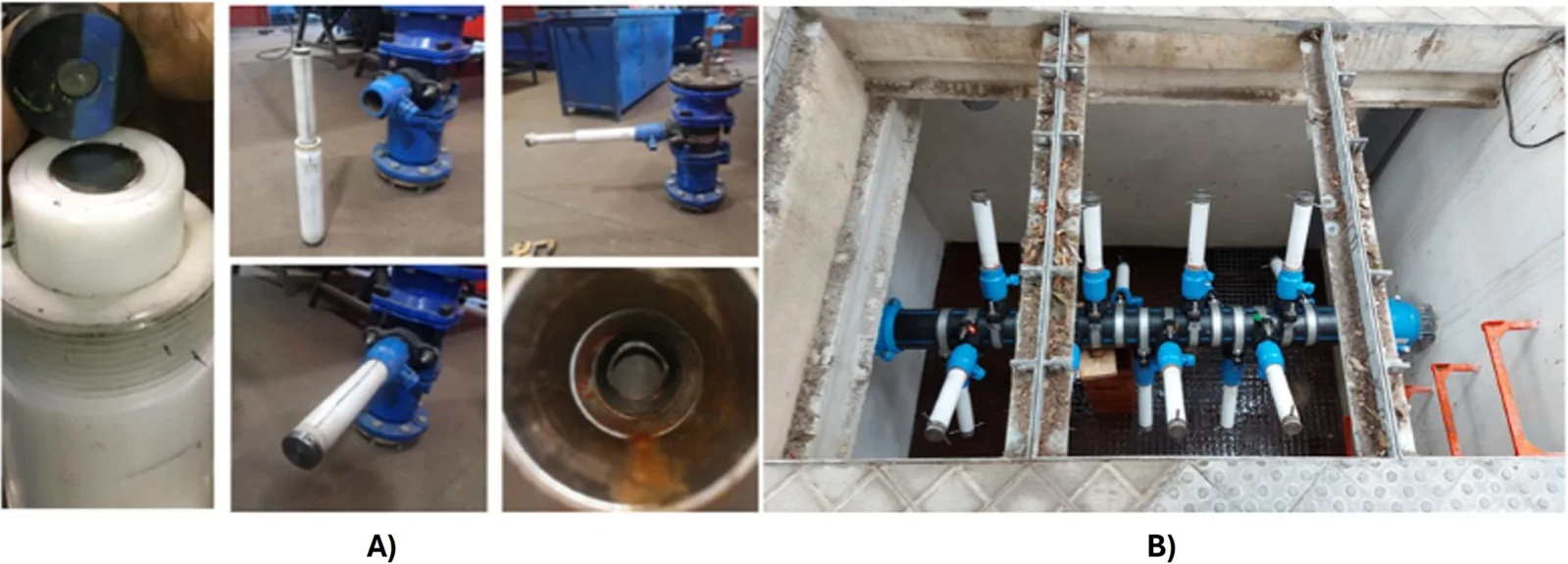
(A) individual coupons installed in the biofilm sampling device, (B) biofilm sampling device with 16 coupons installed in the city of Valencia

**Fig. 2:**
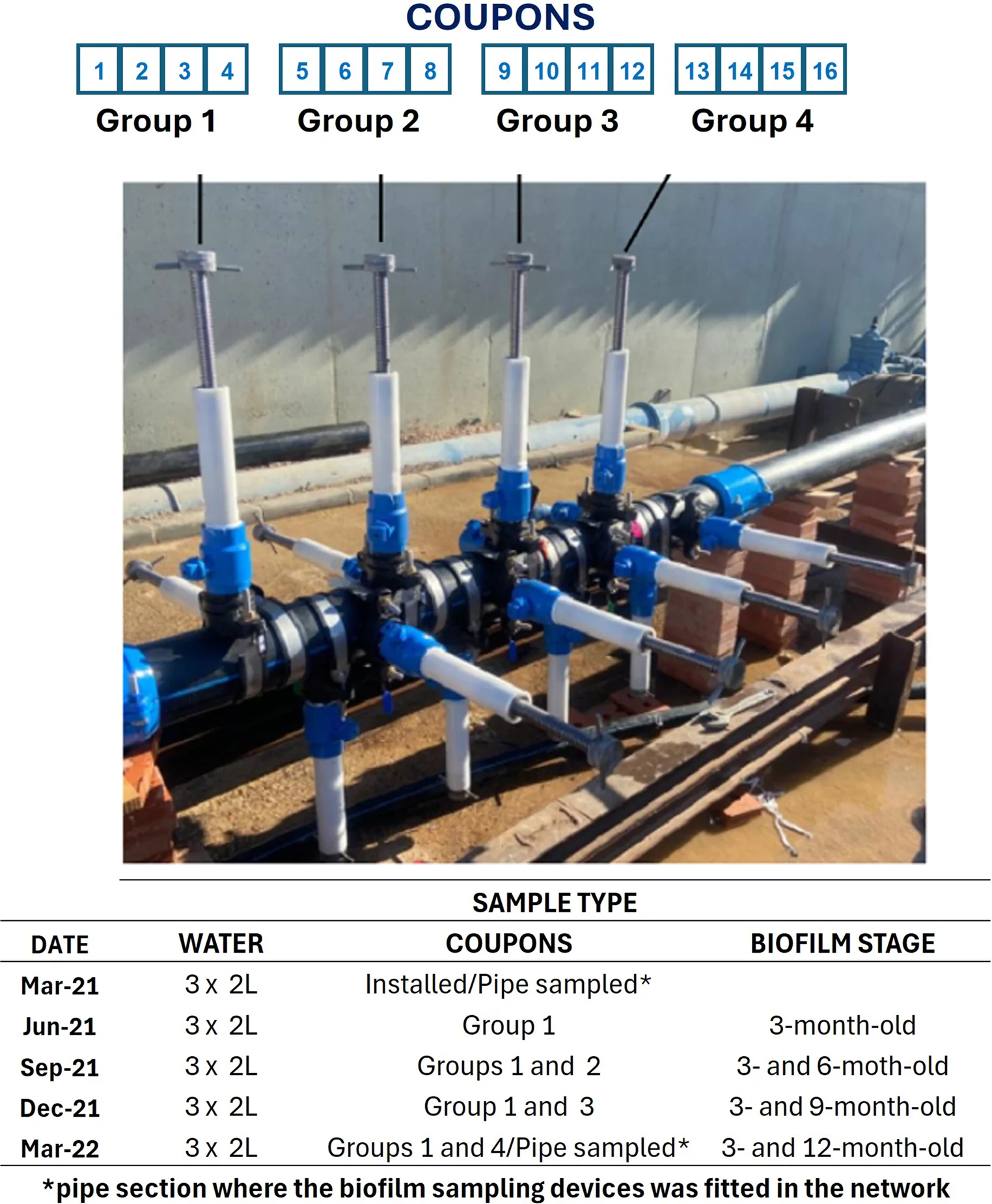
Coupons numbered in blue (1–16) from 1 to 16 installed in the Valencian water network, showing the corresponding sampling group (1–4) and type of sample collected

Biofilm sampling devices (Fig.1) containing 16 coupons made of High-Density Polyethylene (HDPE) were installed in a real chlorinated DWDS made of the same material in the City of Valencia (Spain). Each coupon (Fig. 1A), 5 cm² in size, is placed on a magnetised support held by a telescopic rod that can be rotated by means of a gear mechanism. The installation that allows the coupon to be placed in its original position and subsequently extracted for sampling consist of an under-pressure tapping connection with an intermediate isolation valve. The device was fitted into a 2 m long section of the network (Fig. 1B), and it has the advantage that sampling can be carried out without interrupting the network service, cutting, scraping or flushing the pipes to obtain biofilms. Using these coupons, two different processes were studied: (1) quarterly biofilm re-growth and (2) biofilm succession over a one-year period. The sampling devices were first installed in March 2021 and monitored until March 2022. Every 3 months, the same four coupons were replaced with sterile coupons to study biofilm re-growth dynamics at different seasons starting from a completely clean coupon surface (Fig. 2). For the succession study, three additional groups of four coupons were sampled after 6, 9 and 12 months to study biofilm variation over time (i.e. bacterial long-term succession). Bulk water samples from the supply network (2 L in triplicate) were collected whenever the site was visited for the above coupon collection to compare the two types of habitats (planktonic and biofilm). It should be noted that throughout the studied period there was no other disturbance of the pipeline other than due to hydraulic changes, therefore the biofilm on the inner pipe surfaces was not disturbed. Coupons were removed from the pipe section without stopping the flow through the pipe at different times depending on the sampling group (i.e. quarterly or long-term succession) to which they belonged (Fig. 2). At the time of installation (March 2021) and removal (March 2022) of the biofilm sampling device, a sample of the biofilm already existing in the pipe connecting the device to the main network was obtained for comparison purposes. Taxonomic data in relation to the sequencing of these samples can be found in Supplementary Material (Figures S1 and S2).

Biofilms were removed from the coupons by a standardised brushing method (Deines et al. 2010) to obtain a biofilm suspension in Phosphate-Buffered Saline (PBS) (Sigma-Aldrich, UK) and biomass was concentrated on 0.22-µm nitrocellulose membrane filters (Millipore, Corp.) for subsequent DNA analysis as previously explained (Douterelo et al. 2013). The membranes were dispatched in ice to the University of Sheffield (UK), where the DNA extraction process was performed within 24 h to isolate the genetic material of the biofilm. For the molecular analysis of water samples, three replicates of 2 L per site and sampling event were filtered through 0.22-µm nitrocellulose membrane filters (Millipore, Corp.). DNA from biofilm and bulk water samples were then preserved in the dark at − 80 °C until sequencing analysis.

### Water quality analysis

The water source supplying the network is surface water, and the treatment process consists of initial treatment (screening), followed by primary disinfection using chlorine dioxide and pH adjustment with carbon dioxide. Adsorption using powdered activated carbon is applied to reduce micropollutants, while coagulation and flocculation promote particle aggregation and subsequent removal through sedimentation. The clarified water is then subjected to filtration through granular activated carbon to remove residual particulates and dissolved organic matter. Final disinfection is achieved through UV irradiation and chlorination to ensure compliance with European drinking water quality standards (EU Directive 2020/2184).

On the dates of coupon collection, samples from the water that supplied the network were collected via sampling taps located immediately upstream of the biofilm sampling devices. The water quality analysis covered a wide range of physical and chemical parameters, all data is shown in Supplementary Material Table S1, including chorine, geosmin, metals and DBPs, and was performed following standard methods by the Empresa Mixta Valenciana del Agua, S.A. (Spain). All the analyses were carried out following relevant regulatory water quality standards and criteria established for drinking water quality in Spain (Real Decreto 3/2023), the EU Directive (Directive 2020/2184) and regional legislation (Decreto 58/2006 Comunidad Valenciana). These standards outline parameters, limits, and monitoring frequencies for both chemical and microbiological agents to ensure tap water is safe to drink. Conventional and regulated microbiological parameters, using culture-based methods were carried out including the detection of FIB and pathogens such as *Escherichia coli*,* Salmonella spp.* and *Clostridium perfringens* (Table 1). The presence of moulds and yeast was also analysed in the samples.

### DNA extractions and amplicon sequencing of the 16S rRNA gene

DNA was extracted from biofilm (*n* = 30), and water samples (*n* = 15) using a method based on proteinase K digestion and cetyltrimethylammonium bromide (CTAB) followed by further DNA purification using phenol/isoamyl alcohol protocol (Neufeld et al. 2007). Quantity and purity of the extracted DNA were assessed using NanoDrop ND-1000 spectrophotometer (NanoDrop, Wilmington, DE, USA). The DNA quality ratio was evaluated by absorbance at 260/280 nm, DNA outside the ∼1.7 was concentrated and cleaned using the Wizard^®^ DNA Clean-Up System (Promega, UK).

Sequencing was performed at Mr DNA Laboratory (Shallowater, TX, USA) on the Illumina MiSeq platform following the manufacturer’s guidelines for pair-end sequencing. The 16S rRNA gene of bacteria was amplified using the primers 28F (GAGTTTGATCNTGGCTCAG) and 519R (GTNTTACNGCGGCKGCTG) spanning the V1 to V3 hypervariable regions. Briefly, 30 PCR cycles were performed using the HotStarTaq Plus Master Mix Kit (Qiagen, USA) and the following protocol: 94 °C for 3 min, followed by 30 cycles of 94 °C for 30 s, 53 °C for 40 s, 72 °C for 1 min, and a final elongation step at 72 °C for 5 min. PCR products were checked after the amplification in 2% agarose gel to determine the success of amplification and the relative intensity of bands. Raw sequences were subjected to quality control to evaluate the number, the quality, and the length of the initial reads using a pipeline developed by Mr DNA Laboratory (Douterelo et al. 2020a, b). Subsequently, sequences were imported into the Quantitative Insights into Microbial Ecology 2 program (QIIME2, version 2019.7, qiime2.org) (Bolyen et al. 2019). QIIME2, was used to join pair-end sequences, dereplicate them, identify and filter chimeric sequences, and make *de-novo* clustering by 97% similarity to obtain the operational taxonomic units (OTUs).

### Statistical analyses

All statistical analyses were conducted in R (R Core Team 2024). Microbial community data were analysed using relative abundance matrices at class and genus taxonomic levels. Microbial diversity was assessed using several ecological indices that describe different aspects of community structure. The Shannon–Wiener index (H’) (Shannon 1948; Willis 2019) and Simpson index (1–D) (Simpson 1949; Willis 2019) were calculated to evaluate community diversity and evenness. Species richness was determined using the number of observed taxa, and the Margalef index (Margalef 1958) was calculated to provide a richness-based diversity metric. The Berger–Parker index (Berger and Parker 1970) was used to estimate dominance within the microbial communities. These indices were calculated using the *vegan* package in R (Oksanen et al. 2022). The Shannon and Simpson indices were computed using the diversity() function, while species richness was obtained using the specnumber() function. The Margalef index was calculated based on species richness and total abundance, and the Berger–Parker dominance index was calculated using a custom function. Differences in microbial richness among sampling periods were evaluated using analysis of variance (ANOVA) followed by Tukey’s post hoc test when significant differences were detected.

Community dissimilarity matrices were calculated using Bray–Curtis distances (Bray and Curtis 1957) and based on these distances a non-metric multidimensional scaling (NMDS) was performed using the metaMDS() function from the vegan package to visualise similarities and differences in microbial community composition among samples. Principal component analysis (PCA) was used to identify dominant gradients in microbial community structure across samples. To evaluate differences in microbial community composition between bulk water and biofilm samples, permutational multivariate analysis of variance (PERMANOVA) was performed using the adonis2() function with 999 permutations. To ensure that PERMANOVA results were not influenced by differences in dispersion among groups, homogeneity of multivariate dispersion was assessed using the betadisper() function.

To investigate biofilm development over time, microbial community composition was also analysed across biofilms of different ages (3, 6, 9 and 12 months). Multivariate analyses, including NMDS, PCA, PERMANOVA (Anderson 2001), and homogeneity of multivariate dispersion (PERMDISP), were used to evaluate temporal changes in community structure and to assess patterns of microbial succession during biofilm maturation.

Relationships between microbial community patterns and physicochemical parameters were explored using Pearson correlation analysis. The correlation matrices were calculated using the cor() function, and the significance of correlations was established using a p-value threshold of 0.05.

## Results

### Water quality analysis

The key results of the physicochemical and microbiological analyses are described in Table 1, and all the water quality analyses with all the results for the parameters analysed, can be found in Supplementary Material Table S1. Results from the culture-based microbiological analysis do not show the presence of FIB and pathogens in any of the samples. Overall, several of the physicochemical parameters analysed to test for drinking water quality such as alkalinity, pH, turbidity, conductivity, dissolved organic carbon and total organic carbon remained stable over time. An increase in temperature in June (up to 20.6 °C) and September (up to 25.4 °C) was observed coincident with an increase in DBPs such as Trihalomethane (THMs) that reached a maximum level of 48 µg/L. The DBPs (bromodichloromethane, chlorodibromomethane, tribromomethane and trichloromethane) increased with higher temperatures and chlorine dosing in the system (> 0.5 mg/L Cl_2_ in June and September, Supplementary Table 1). Turbidity remained below 1 NTU in all samples, indicating good water quality and remaining well below the Spain regulatory limit of 4 NTU from drinking water (Gobierno de España, 2023). Iron and manganese levels, typically associated with water discolouration processes (Douterelo et al. 2014), were higher in March and September when compared with other months.

**Table 1:** Physicochemical and microbiological parameters of water samples collected across different quarters

Parameters		2021				2022
		March	June	September	December	March
Physico-chemical	Free residual chlorine (mg/L)	0.54	0.95	0.75	0.72	0.95
	Temperature ("C)	14.8	20.6	25.4	15.3	14.2
	Dissolved aluminium (ug/L)	<50	87	71	69	110
	Dissolved calcium (mg/L)	123	122	108	108	110
	Sulphates (mg/L SO_4_)	248	260	247	257	218
	Total Alkalinity (mg/L CaCO_3_)	220	220	160	200	220
	pH	7.8	7.7	7.8	7.7	7.7
	Conductivity (uS/cm)	970	980	910	930	850
	Turbidity (NTU)	0.4	<0.3	<0.3	<0.3	<0.3
	Iron (ug/L Fe)	52	<2	60.4	9	11.2
	Manganese (ug/L Mn)	1.9	<1	9.3	1.4	25.5
	Sum of THMs (ug/L)	21	28	48	20	25
Microbiological(CFU/100 mL)	*Legionella* spp.	No	No	No	No	No
	*Clostridium perfringens*	0	0	0	0	0
	*Serratia* spp.	Absent	Absent	Absent	Absent	Absent
	*Salmonella* spp.	Absent	Absent	Absent	Absent	Absent
	*Staphylococcus aureus*	0	0	0	0	0
	Molds and yeasts	<1	<1	<1	<1	<1
	Total coliforms	0	0	0	0	0

Note: 0 = no colonies detected by quantitative culture-based methods; No/Absent = target organism not detected by presence-absence methods; <1= below the analytical detection limit of the method

Pearsons’s correlation analyses (Supplementary Material Table S2) were carried out between different physicochemical parameters and temperature. The results showed significant correlations between temperature and THMs, pH, and metal concentrations. Other parameters despite of being associated did not show significant statistical correlations.

### Comparison between biofilm and bulk water bacterial communities

**Fig. 3:**
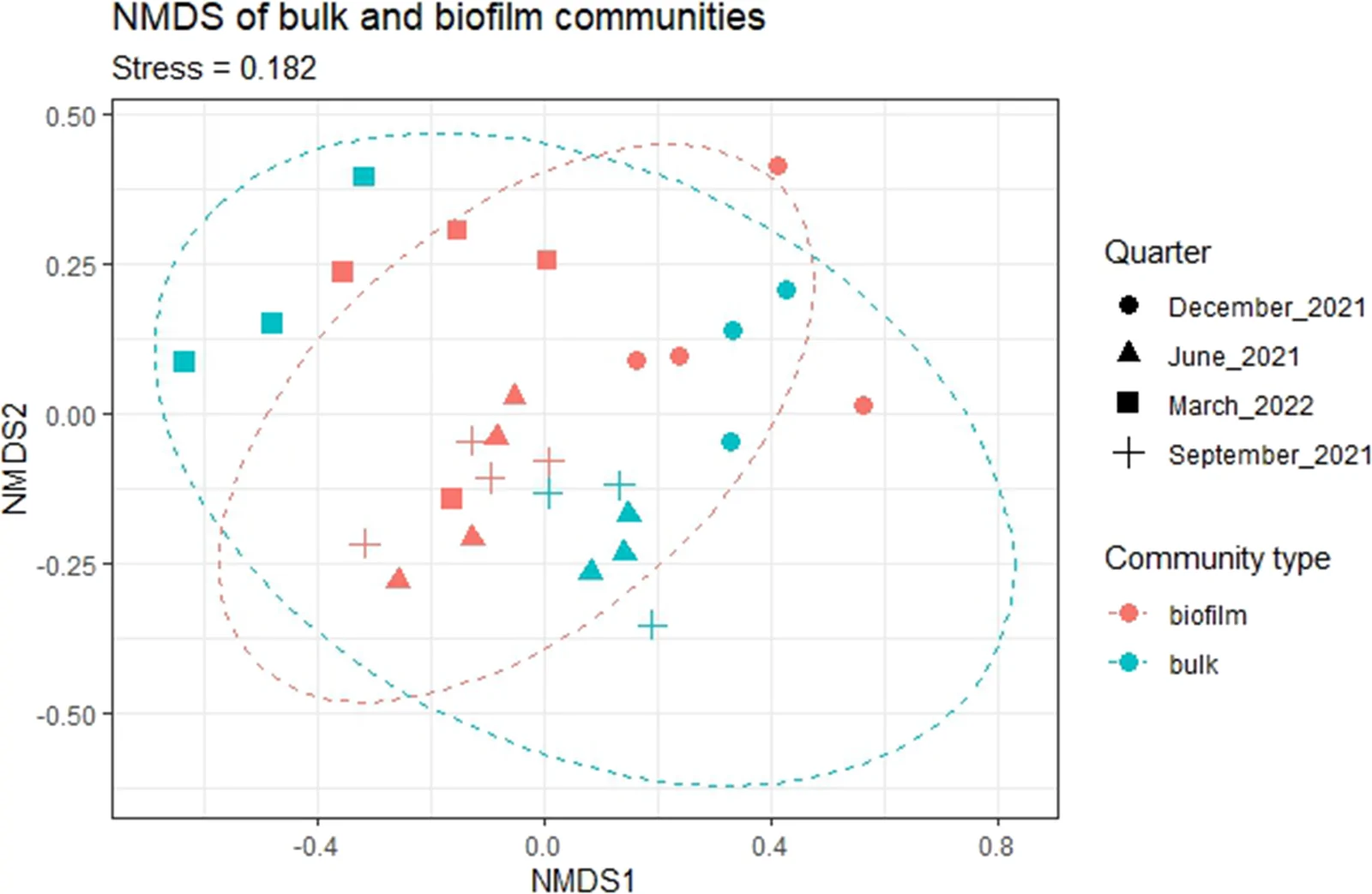
NMDS ordination based on Bray-Curtis dissimilarities. Calculated from the relative abundances of the four dominant bacterial classes in biofilm and bulk water samples. All the biofilm samples analysed are from 3-month-old biofilms

At class level, bulk water samples were dominated by Alphaproteobacteria from March 2021 to December 2021, reaching relative abundances of up to 63%, while in March 2022 Actinomycetia became the dominant class, representing more than 45% of the relative abundance in all samples (Supplementary Material Fig. S1). Biofilm communities were generally dominated by Betaproteobacteria. The greatest variation was observed in the 12-month-old biofilms, where most replicates remained dominated by Betaproteobacteria (~ 85% relative abundance), followed by Alphaproteobacteria (~ 16%). However, one replicate showed a contrasting community structure and was dominated by Gammaproteobacteria (79% relative abundance) (Supplementary Material Fig. S1). For comparative reasons biofilm samples from the pipe where the biofilm sampling device was connected were sampled at the beginning of the monitoring period (March 2021, sample ID: PSI) and end (March 2022, sample ID: PSM). The initial sample (PSI) showed a similar composition to 3-month-old biofilm, showing the influence of the microbial community already colonising the pipe. However, sample PSM showed a more diverse community than the biofilm sample obtained from the network, with higher relative abundance of Gammaproteobacteria, Actinomycetia and Bacilli. To further understand differences in microbial community structure between bulk water and biofilm samples, an NMDS analysis was performed using Bray-Curtis dissimilarities calculated from the relative abundances of the four dominant bacterial classes (Fig. 3). The dashed ellipses represent the dispersion of samples within each community type, highlighting the tendency of biofilm and bulk water samples to form distinct clusters. The NMDS stress value (0.182) indicates an acceptable representation of the data in two dimensions. The PERMANOVA analysis confirmed that both sample type (biofilm vs. bulk water) and sampling period significantly influenced microbial community composition (Type: R² = 0.128, F = 5.789, *p* = 0.001; Quarter: R² = 0.365, *p* = 0.001). However, the variation explained by sample type was relatively modest compared with the effect associated with sampling period. The test for homogeneity of multivariate dispersion was not significant (betadisper, *p* = 0.9).

Results at genera level for both water and biofilm samples can be found in Supplementary Material Figures S2 and S3. Water showed a clear seasonality with percentages of main bacterial genera changing over time, with March 2021 samples including *Brevundimonas* (4–17%) and *Chryseobacterium*, June samples containing *Rhodocyclus* (22–35%), *Reyranella* (10–12%) and *Anabaena* (9–16%). September with *Flavobacterium* (19–33%) and *Brevundimonas* (15%), and December with higher *Pseudoclavibacter* (7–20%). The final water samples collected (March 2022), showed very high abundance of *Corynebacterium* (25–75%). The taxonomy at genera level of all biofilm samples can be found in Supplementary Material Fig. S3 and is discussed in more details in next sections. In summary, biofilms samples were dominated by *Acidovorax* in 3- and 12-month-old biofilms, showing higher diversity in 6- and 9-month-olds biofilms.

### Environmental drivers of bacterial community composition: water vs. biofilm

**Fig. 4:**
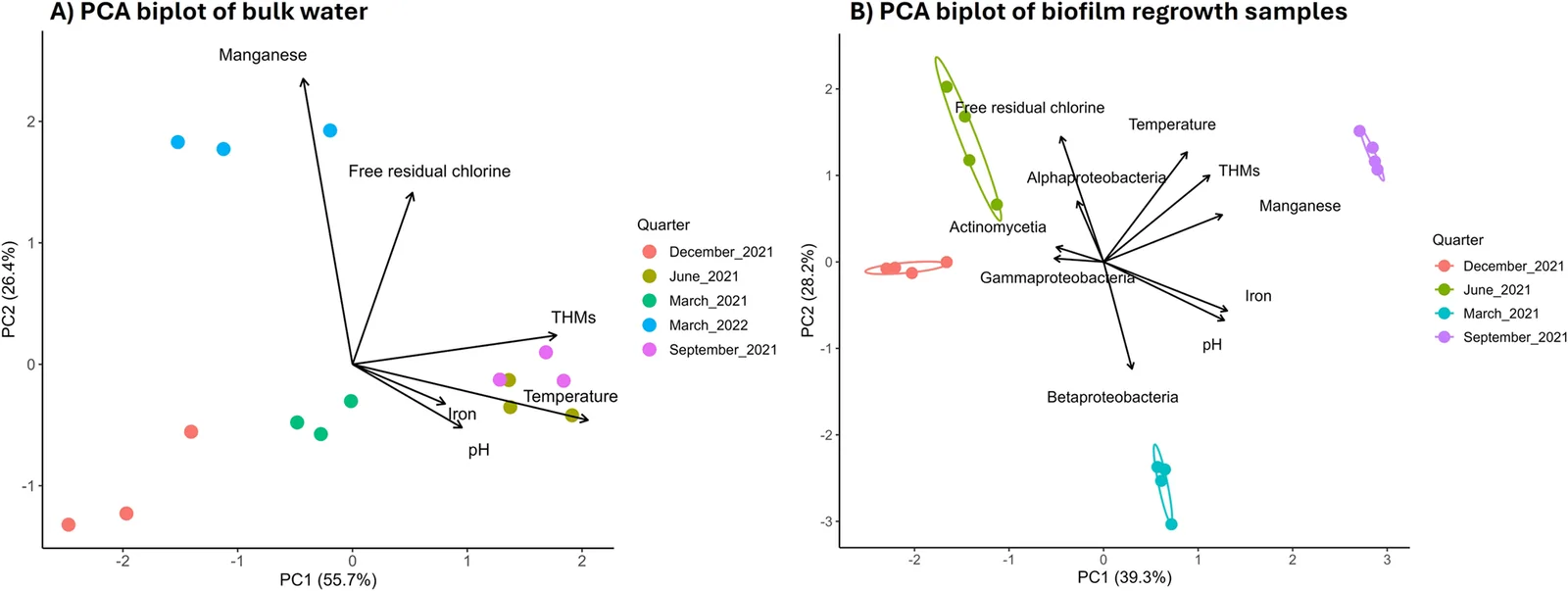
PCA biplots: (**A**) of bulk water bacterial and (**B**) biofilm regrowth samples. Arrows indicate the direction and strength of environmental variables influencing community composition

To understand the relationship between bacterial community composition and physicochemical parameters in water samples, a PCA was performed based on class-level relative abundances and selected environmental variables (Fig. 4A). For water samples, the first principal component (PC1) explained 55.7% of the total variance, while the second principal component (PC2) explained 26.4%, together accounting for over 80% of the variability in the dataset. The distribution of samples along the PCA axes showed clear differences between sampling periods, indicating temporal variation in water microbial communities. Samples from June and September 2021, were associated with positive values of PC1 and aligned with higher contributions of THMs and temperature. June 2021 samples also showed some association with pH and iron. In contrast, samples from March and December 2021 were positioned towards negative PC1 values. March 2022 samples were separated along PC2 and were located closer to manganese and free residual chlorine. Iron and pH were also related to sample distribution, mainly on the positive side of PC1. Overall, samples collected during warmer months were positioned in the same area of the ordination as temperature and THMs, whereas samples from cooler periods occupied different regions of the plot. Similar patterns linking microbial communities with water chemistry have been described in DWDS (Dai et al. 2020). In a recent study, Leifels et al. (2025) reported that changes in hydraulic conditions promoted biofilm detachment and were accompanied by shifts in bulk water microbial communities. Taking these results into account, it is possible to observe that samples collected in different seasons occupied distinct positions in the PCA ordination. In particular, samples associated with higher temperatures and THMs concentrations were clearly separated from those collected during cooler periods, suggesting that temporal changes in water quality may be associated with differences in bacterial community composition.

The PCA for biofilm samples (Fig. 4B) showed that the PC1 explained 39.3% of the total variance, while the PC2 explained 28.2%, together accounting for a substantial proportion of the dataset variability. The ordination showed a clear separation of samples according to sampling period, indicating temporal shifts in biofilm bacterial communities. Samples from September 2021 were strongly associated with positive values of PC1 and aligned with higher contributions of temperature, THMs, and manganese. Whereas, June 2021 samples showed a stronger association with free residual chlorine and Alphaproteobacteria. March 2021 samples were associated with Betaproteobacteria and December 2021 samples were more closely related to Gammaproteobacteria and Actinomycetia. The PCA shows that temporal variation in biofilms was associated with changes in water chemistry such as temperature, DBPs, and residual chlorine. These results suggest that, similarly to bulk water, physicochemical conditions play an important role in shaping biofilm community structure, although those changes were stage-dependent during biofilm maturation.

### Biofilm community composition: temporal succession and core microbiome

**Fig. 5:**
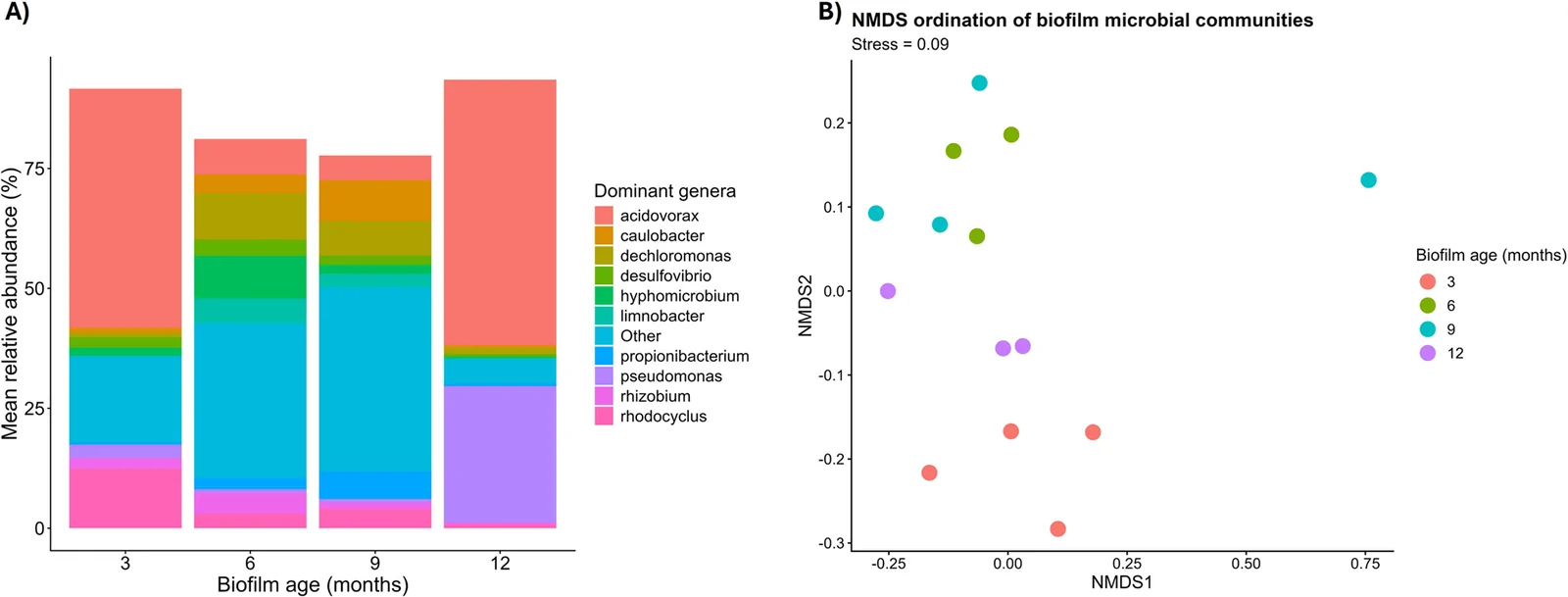
Biofilm bacterial re-growth showing: (**A**) Mean relative abundance of dominant genera in biofilms at different stages of development (3, 6, 9 and 12 months) and (**B**) NMDS showing temporal shifts in microbial community composition

Taxonomic analysis was performed at the genus level to evaluate bacterial changes during biofilm succession (3-, 6-, 9- and 12-months old biofilms). Figure 5A shows the analysis with the most dominant genera, further information on diversity at genera level is included in the Supplementary Material Figure S3. Several dominant genera were identified, including *Acidovorax*, *Pseudomonas*, *Caulobacter*, *Rhizobium*, *Dechloromonas*, and *Desulfovibrio* (Fig. 5A). However, the relative abundance of these dominant genera varied across biofilm age, indicating temporal shifts in community composition during biofilm development. In early-stage biofilms (3 months), the bacterial community was dominated by *Acidovorax* and members of the *Rhodocyclus* group, whereas older biofilms (> 3 months old) showed a greater contribution of genera such as *Pseudomonas*, *Dechloromonas*, and *Caulobacter*.

To further investigate temporal changes in bacterial communities during biofilm development, an NMDS analysis was performed considering samples collected at different stages (3, 6, 9 and 12 months). The ordination showed a separation of samples according to biofilm age (Fig. 5B), indicating temporal shifts in community composition during biofilm maturation.

PERMANOVA analysis confirmed that biofilm age significantly affected bacterial community structure (R² = 0.447, F = 2.700, *p* = 0.001). In addition, the test for homogeneity of multivariate dispersion was not significant (betadisper, *p* = 0.709), indicating that the observed differences among age groups were not driven by unequal dispersion. Overall, these results support progressive changes in microbial assemblages as the biofilm matured over time.

**Fig. 6:**
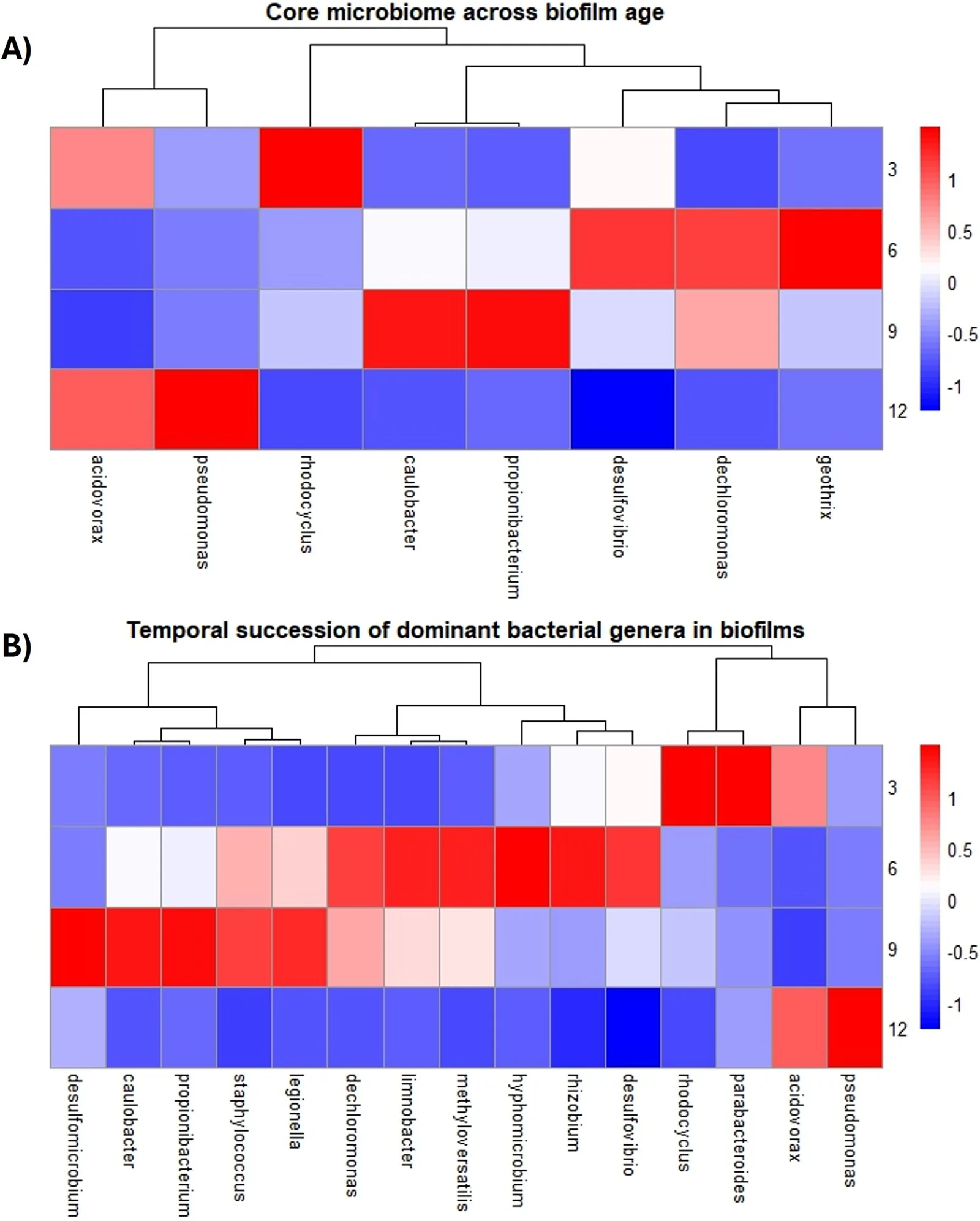
Heatmaps showing the relative abundance at genera level of (**A**) the core biofilm microbiome and (**B**) biofilm maturation stages (3, 6, 9 and 12 months). Colour intensity reflects scaled abundance values, with red indicating higher relative abundance

To identify the biofilm core microbiome (i.e. persistent members of the bacterial community over time), analysis at genera level was performed considering all biofilm maturation stages (3-, 6-, 9- and 12-months samples) (Fig. 6A). A total of 8 genera met this criterion, being consistently detected across all samples, indicating the presence of a stable core community throughout biofilm development. The core microbiome included genera such as *Acidovorax*,* Rhodocyclus*,* Desulfovibrio*,* Dechloromonas*,* Geothrix*,* Pseudomonas*,* Caulobacter *and* Propionibacterium.* These taxa were present across all time points, yet their relative abundances varied during biofilm maturation stages. For example, *Acidovorax* showed higher relative abundance in early (3 months) and late stages (12 months), whereas genera such as *Dechloromonas* and *Geothrix* showed higher relative abundance during intermediate stages (6–9 months). These patterns indicate that, despite the persistence of core taxa, their relative contributions change over time.

When the temporal succession of dominant bacterial genera was analysed (Fig. 6B), genera belonging to *Parabacteroides* and *Rhodocyclus* dominated the composition of 3-month-old biofilms, while 6-month-old biofilms were dominated by *Dechloromonas*, *Hypomicrobium*, *Rhizobium* and *Desulfovibrio*. At the latest stages of biofilm development, 9-month-old biofilms were dominated by *Desulfomicrobium*, *Caulobacter* and Propionibacterium, and the relative abundance of potential pathogenic bacteria such as *Legionella* and *Staphylococcus* increased. Samples from the last biofilm stage, 12-month-old, have two main genera *Pseudomonas* and *Acidovorax* dominating the composition of the biofilms.

### Comparison of biofilm characteristics over time using diversity indices

**Fig. 7:**
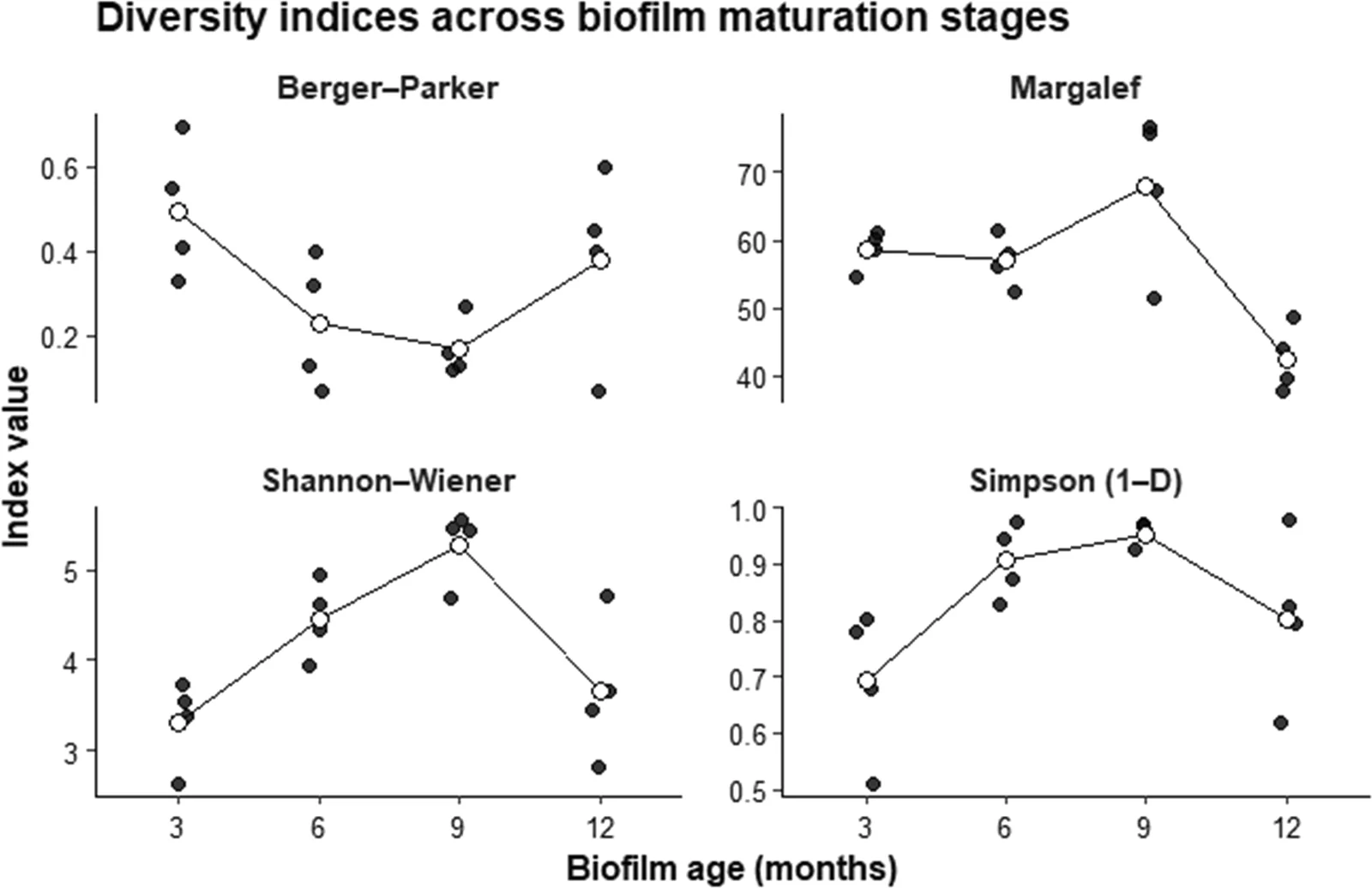
Diversity indices across biofilm maturation stages (3, 6, 9 and 12 months). Points represent individual samples and open circles connected by lines indicate mean values for each stage

Clear temporal patterns were observed in biofilm diversity indices across maturation stages (Fig. 7). The Berger–Parker index decreased from 3 to 9 months, indicating a reduction in dominance during early biofilm development, followed by an increase at 12 months. Consistent with this pattern, both Shannon–Wiener and Simpson (1–D) indices increased from 3 to 9 months, reflecting a progressive rise in diversity and evenness, and subsequently declined at 12 months. The Margalef index was relatively stable over time, with a small peak at 9 months and lower values at 12 months. Biofilm communities reached maximum diversity and structural balance at intermediate stages of maturation, followed by a shift towards increased dominance and reduced diversity in more developed biofilms.

## Discussion

### Impact of physicochemical parameters on water quality and safety

Water temperature was the main factor affecting water quality (Table 1). The period between June and September, when temperatures exceeded 20 °C, coincided with a clear decrease in chlorine residual. Similar reductions in chlorine residual associated with elevated temperatures have been reported in other drinking water systems (Garcia-Ávila et al., 2020) and may reduce disinfection efficiency by allowing greater microbial activity and biofilm growth within DWDS (Kalita et al. 2024; Fish and Boxall 2018). The main concern in relation to this disinfectant reduction is the potential proliferation of pathogens that might survive in biofilms. No pathogenic species and FIB were found in water samples using conventional regulated methods (i.e. culture-based methods), however the sequencing analysis showed higher relative abundance of potential opportunistic pathogenic bacteria such as *Legionella* spp. and *Staphylococcus spp.* in 9-month-old biofilm samples. Controlled experimental studies in chlorinated DWDS have previously demonstrated that elevated temperatures enhance the growth of opportunistic pathogens, including *Legionella pneumophila*, *Mycobacterium avium* complex, and *Pseudomonas aeruginosa*, relative to the average temperatures observed in UK networks (Calero et al. 2021). These microorganisms are of particular concern because they can persist within biofilms, resist disinfectants, and in some cases proliferate inside amoeba (Del Olmo et al. 2021; LeChevallier et al. 2024), increasing the risk of their release into bulk water.

We also observed that DBPs formation, particularly THMs concentrations, increased with higher temperatures, reaching their highest values in September (25.4. °C). Despite the observed DBPs level rise their concentrations were never above the regulatory limit. DBPs commonly formed from reactions between disinfectants and natural organic matter, are of concern due to their potential carcinogenic and toxic effects (Wang et al. 2025). DBPs formation in DWDS is not only influenced by temperature, but also dependent of pH, organic carbon content, type of disinfectant and dose, and water age (Montoya-Pachongo et al. 2018; Valdivia-Garcia et al. 2019). Previous studies in drinking water systems have shown that higher temperatures increase the rate of reactions between disinfectants and natural organic matter, leading to DBPs formation (Zhang et al., 2012; Garcia-Ávila et al., 2020; Kalita et al. 2024). Our results reinforce that maintaining an adequate disinfectant residual is essential to control biofilm-associated pathogens, but must be balanced against the DBPs formation, particularly under elevated temperatures (Lee et al. 2023).

When both PCA analyses were considered together, a similar temporal pattern can be observed in bulk water and biofilm samples. Although the microbial composition of the two habitats was different, both showed shifts associated with temperature, THMs and residual chlorine. This suggests that changes in water quality may influence both planktonic and biofilm communities within the distribution system (Dai et al. 2020; Chen et al. 2025). Biofilm and bulk water communities were clearly different, as also shown by the NMDS and PERMANOVA analyses. However, both followed comparable temporal patterns and both were associated with the same physicochemical variables. This indicates that although the two habitats contain different bacterial assemblages, they may respond to the same environmental drivers within the distribution system. These observations suggest a strong connection between water chemistry, biofilm development and the microbial community present in bulk water. Therefore, changes in water quality parameters may influence both habitats simultaneously (Leifels et al. 2025; Wingender and Flemming 2011).

Likewise, we detected higher iron and manganese concentrations during warmer period (Table 1). The increase of iron and manganese concentrations, could be partly related to temperature-driven processes, such as changes in redox conditions, increases in microbial activity and biofilm growth (Kalita et al. 2024; Fish and Boxall 2018). Ginige et al., 2011 studied the contribution of pipe wall biofilms towards iron and manganese deposition via reactors and field work tests and observed that temperature affected biofilms growth and subsequently deposition of these two metals. Other factors may also have played a role in the increase of these metals, since small hydraulic disturbances in the network can resuspend accumulated material from sections of cast iron pipes located upstream where the biofilm sampling device was installed, leading to temporary increases in metal concentrations in the water. Taken together, these processes increase the risk of water discolouration and other aesthetic issues that commonly trigger consumer complaints (Douterelo et al. 2019; Sunny et al., 2020).

### Bacterial community diversity in biofilm samples: succession and maturation

The biofilm community composition changed over time, showing that temporal dynamics and long-term succession occur even under relatively stable network operational conditions. Biofilm age was a key factor shaping bacterial community structure, indicating that biofilms undergo ecological succession. This succession is characterised by shifts in relative abundance of dominant taxa (i.e. core microbiome) and increasing structural complexity due to less abundant taxonomic groups. Previous studies in chlorinated systems have also shown biofilm succession over time at different time scales (Martiny et al. 2003). Nonetheless, when biofilm reaches a mature state and compositional stability in DWDS is still not clear, and full community stabilisation, if ever achieved, may take years (Flemming et al. 2016). Chen et al. 2025 showed in a pilot DWDS that biofilm development over 56 weeks was influenced by type of disinfectant and that biofilms growing under free chlorine tended to be more stable than those with no chlorine or with chloramine. We previously observed in a chlorinated DWDS test facility that biofilm structure did not reach stability after 3 months of development (Douterelo et al. 2020a, b). Recent studies also suggest that although a biofilm may be considered relatively mature after approximately 8–9 months of development (Søborg et al. 2024), it should not be regarded as a static community. Minor changes in community composition may still occur over time in response to environmental conditions (Erdei-Tombor et al. 2024). Similarly the present study in a chlorinated network observed that biofilm structure changed as maturation proceeded, involving initial colonisation by certain bacteria and subsequent incorporation of others. Overall, these results suggest that biofilms become more complex as they grow and their development is not completely linear, with intermediate stages showing the highest diversity at 9 months (Fig. 7), and later stages (12 months) becoming dominated by less taxa, mainly *Pseudomonas* and *Acidovorax*, but still changing.

For contextual comparison, biofilm samples were collected directly from the pipe wall at the beginning (PSI) and end (PSM) of the monitoring period. PSI and the younger coupon biofilms shared dominant taxonomic groups, although differences in relative abundance were observed. This suggests that the coupons were colonised by bacterial groups already present in the distribution system. The presence of these shared dominant taxa between coupon and pipe-wall biofilms suggests that the *in situ* devices capture representative members of the microbial community present in the distribution system and provide a useful approach for studying biofilm dynamics under operational conditions. Interestingly, PSI and PSM also differed in community composition despite both representing biofilms established on the same pipe section. Differences observed between PSI and PSM indicate that the microbial composition of pipe-wall biofilms changed during the monitoring period, suggesting that these communities may not remain completely stable over time. Similar temporal variability has previously been reported in drinking water biofilms, where environmental conditions and operational factors can influence microbial succession even in established biofilms (Martiny et al. 2003; Fish and Boxall 2018; Flemming et al. 2016).

The increase in diversity observed at 6- and 9-month-old biofilm samples can be related to the formation of new niches within the more mature biofilm, allowing microorganisms with different metabolic requirements to coexist (Flemming et al. 2016; Battin et al., 2016). Betaproteobacteria was the dominant class in younger biofilms (3 months) and at genera level *Rhodocyclus*, *Parabacteroides* and *Acidovorax* were the main represented genera. *Rhodocyclus spp.* is a common resident of DWDS and has been related to iron-related issues (i.e. “red water” events) (Li et al. 2010). The presence of this type of bacteria indicates the potential for water discolouration events if the biofilm dislodges from the pipe walls and is mobilised in the water. *Acidovorax* is a common inhabitant of DWDS associated to Fe/Mn oxidation (Vandermaesen et al. 2017), while *Parobacteroides* is a gut-associated anaerobic microorganism not commonly found in DWDS, that could indicate a potential contamination event in the network.

Later biofilm stages, 6- and 9-month-old biofilms tended to include a wider range of taxa with a higher contribution from other genera such as *Desulfomicrobium*,* Caulobacter*,* Propionibacterium*,* Staphylococcus* and *Legionella.* Several of these *g*enera, commonly reported in drinking water systems, include *Caulobacter*, *Acidovorax*, and *Legionella*, which are associated with biofilm formation and pathogen persistence (Margot et al. 2024), whilst other taxa such as *Desulfomicrobium*,* Staphylococcus*, and *Propionibacterium* may occur in anaerobic niches or as opportunistic contaminants. Sulfate-reducing bacteria such as *Desulfomicrobium* are associated with anaerobic environments and corrosion processes in drinking water biofilms (Gomez-Smith et al. 2015) while *Staphylococcus* and *Propionibacterium* have been detected in DWDS, typically present in low abundance and as opportunistic members of the microbiome (Dai et al. 2020; Moreno et al. 2024). At 12 months, biofilm diversity decreases, showing that one or a few bacterial groups become more dominant, including *Acidovorax* and *Pseudomonas.* Both genera are commonly detected in drinking water systems, where they contribute to biofilm formation, and in particular *Pseudomonas* is widely recognised for its resistance to disinfection and its role as an opportunistic pathogen (Wingender and Flemming 2011; Douterelo et al. 2014). The reduction in diversity within biofilms, could be due to competition or because some taxa are better adapted to the conditions (e.g. resistance to disinfection) in a more mature biofilm. Overall, these results suggest that biofilm development is not completely linear, with intermediate stages showing the highest diversity, and later stages dominated by fewer and more abundant genera. These differences could be explained by the fact that different taxa are able to perform similar ecological roles, allowing biofilms to maintain its function (Louca et al. 2018). Together, these findings support the idea that biofilm maturation is a key driver of community assembly and that biofilms undergo a progressive ecological succession rather than remaining stable over time (Douterelo et al. 2018; Zhu et al. 2021).

### Water quality and microbial dynamics

Clear relationships between water physicochemical parameters and microbial community structure in water and biofilm samples were observed (Fig. 4). The multivariate analysis revealed that temporal variation in microbial communities was associated with changes in temperature, THMs, and metals. In biofilm, September samples with the higher water temperatures, were strongly associated with THMs and manganese, while June samples were more related to free residual chlorine and Alphaproteobacteria. Alphaproteobacteria have been linked to warmer conditions and increased disinfectant levels in other DWDS (McCoy and VanBriesen 2014).

During the warmer months an increase in certain genera was observed in water samples, these were dominated by *Rhodocyclus*, *Reyranella* and *Anabaena. Anabaena* is a cyanobacteria associated with eutrophication (Douterelo et al. 2004) and often cause musty-earthy odours in water due to compounds like geosmin, which, while usually harmless in themselves, signal a potential algal bloom. While water treatment plants can remove cyanobacterial toxins present in surface water sources, elevated biomass during summer blooms presents treatment challenges for water utilities. These include the release of taste and odour compounds such as geosmin, increased DBP precursors, and enhanced microbial activity and biofilm formation at higher temperatures. In this study, the levels of geosmin were always < 0.005 ug/L in all the samples, yet higher temperature were coincident with higher THMs levels (September 2021). Many countries have developed regulations to limit DBPs concentrations in drinking water principally THMs. The contribution of biofilms to DBPs formation has been studied in DWDS, showing that proteins and amino acids present in biofilms may increase the risk of DBPs formation (Li et al. 2022). Periodic biofilm removal, reduction of organic matter and bromide ion concentrations, and maintenance of high flow rates are effective ways to reduce bacterial DBPs (Li et al. 2022).

We found that higher DBPs levels, occurring in warmer months, were coincident with the presence of *Flavobacterium* and *Caulobacter* in water samples, and *Desulfomicrobium*,* Propionibacterium*,* Dechloromonas*,* Staphylococcus* and *Legionella* in biofilms. *Flavobacterium* is associated with the degradation of natural organic matter, and its metabolic activity can increase the availability of DBPs precursors, contributing to higher DBPs formation. Other bacterial groups identified in this study may also influence conditions within the distribution system. *Desulfomicrobium* has been associated with sulphate reduction and metal cycling processes in anaerobic biofilm environments, including iron and manganese reduction (Moreno et al. 2024). *Dechloromonas* is able to reduce chlorine compounds and its presence may contribute to disinfectant decay (Soon-Thiam et al., 2023). *Legionella spp*. and *Staphylococcus spp*. are well-known opportunistic pathogens (LeChevallier et al. 2024). These results suggest that different bacterial groups may have different implications within DWDS. *Rhodocyclus*, *Acidovorax* and *Desulfomicrobium* have been associated with processes linked to iron and manganese cycling, corrosion, and water discolouration, which may affect the aesthetic quality of drinking water. In contrast, the detection of *Legionella spp.*,* Staphylococcus spp.* and *Pseudomonas spp.* are of greater concern because these microorganisms have been associated with opportunistic infections and may persist within drinking water biofilms (Wingender and Flemming 2011; LeChevallier et al. 2024).

The control, removal and monitoring of pipe wall biofilms still represent an important challenge for DWDS management (Douterelo et al. 2016). Mechanical processes such as air scouring and disinfectants such as chlorine dioxide exhibit high biofilm removal capacity and low toxicity (Fish and Boxall 2018; Oliveira et al. 2024). Nevertheless, biofilms are still a challenge for water companies worldwide, and regular monitoring and cleaning strategies are needed to reduce their presence. In this context, the use of *in situ* biofilm devices provided a representative approach for monitoring microbial dynamics directly within an operational DWDS. This monitoring strategy makes it possible to study biofilms without interrupting the water supply and may avoid underestimating microbial loads associated with attached biofilm communities within DWDS, which commonly occurs with traditional monitoring approaches based only on bulk water sampling (Pick and Fish 2024). Once biofilm development and dynamics are better understood, maintenance strategies and operational interventions such as flushing and cleaning programmes may be better designed.

## Conclusions

This study provides new evidence on the influence of temperature on disinfection efficiency, metal mobilisation, and DBPs formation in chlorinated DWDS in Mediterranean regions. Elevated water temperatures were associated with decreased residual chlorine, potentially compromising disinfection performance and promoting higher microbial activity. Also warmer conditions coincided with increased concentrations of dissolved iron and manganese, with peak levels aligning with the presence of metal-reducing bacteria such as *Dechloromonas* and *Caulobacter.* These results suggest a potential role of biofilms in metal mobilisation. Genera such as *Pseudomonas* and *Acidovorax* were consistently identified as part of a core microbiome, indicating their potential contribution to the stability and resilience of biofilm communities. The warmest period sampled in this study was also the period in which changes in microbial community composition and higher THMs concentrations were observed. These findings highlight the interconnected effects of temperature, disinfectant decay, and microbial dynamics on water quality, emphasising the need to consider regular biofilm monitoring through devices as the one presented in this study. If warmer periods become more common in the future, having information about these patterns may help water companies prepare in advance for similar situations. This may be particularly relevant when considering maintenance work, cleaning programmes or disinfection practices within the distribution network. Although this study was conducted in a Mediterranean DWDS, similar temperature-related processes may occur in other warm or seasonally variable DWDS.

By monitoring biofilms directly in the distribution network, water companies can go beyond routine water testing alone and gain a better understanding of the actual microbial status of the network. Understanding biofilm dynamics can inform disinfection adjustments at the treatment level, as well as network cleaning strategies such as air scouring or ice pigging, helping to prevent water quality problems before the water reaches consumers’ taps.

## Supplementary Information

Below is the link to the electronic supplementary material.

Supplementary Material 1 (PDF 485 KB)

## Data Availability

All data generated and scripts used in this study are publicly available in the Zenodo repository: https://doi.org/10.5281/zenodo.19455769.
