# Supplementary material for: Biofilms and disinfection by-products in Mediterranean drinking water networks: effect of temperature on disinfection strategies
Source: World J Microbiol Biotechnol. 2026 Aug 3;42(8):443. doi: 10.1007/s11274-026-05101-x (PMC13433392; doi:10.1007/s11274-026-05101-x)

SUPLEMENTARY MATERIAL

Table S1. Physicochemical and microbiological parameters of water samples collected across different quarters.

	March 2021	June 2021	September 2021	December 2021	March 2022
Free residual chlorine (mg/L)	0.54	0.95	0.75	0.72	0.95
Temperature (ºC)	14.8	20.6	25.4	15.3	14.2
Dissolved aluminium (µg/L)	<50	87	71	69	110
Dissolved calcium (mg/L)	123	122	108	108	110
Total hardness (ºF)	49	48	43	43	41
Nitrates (mg/L NO_3)	5.4	6.0	3.7	3.8	5.0
Nitrites (mg/L NO_2)	<0.02	<0.02	<0.02	<0.02	<0.02
Sulphates (mg/L SO_4)	248	260	247	257	218
Total Alkalinity (mg/L $CaCO_3$)	220	220	160	200	220
pH	7.8	7.7	7.8	7.7	7.7
Conductivity (µS/cm)	970	980	910	930	850
Turbidity (UNT)	0.4	<0.3	<0.3	<0.3	<0.3
Dissolved organic carbón (mg/L C)	0.8	1.1	1.0	1.3	1.1
Total organic carbón (mg/L C)	0.8	1.2	1.0	1.3	1.2
Iron (µg/L Fe)	52	<2	60.4	9.0	11.2
Manganese (µg/L Mn)	1.9	<1	9.3	1.4	25.5
Fluorides ((µg/L F)	0.32	0.29	0.21	0.25	<1
Langelier Index	0.70	0.64	0.5	0.58	0.62
Bromodichloromethane (µg/L)	3.0	4.1	6.5	3.7	3.0
Chlorodibromomethane (µg/L)	8.6	13	19	8.5	10
Sum of THMs (µg/L)	21	28	48	20	25
Tribromomethane (µg/L)	9.6	11	20	7.0	11
Trichloromethane (µg/L)	<1	<1	1.6	1.1	0.6
Geosmin (µg/L)	<0.005	<0.005	<0.005	<0.005	<0.005
Legionella spp.	No detected	No detected	No detected	No detected	No detected
Clostridium perfringens (cfu/100 mL)	0	0	0	0	0
Serratia spp. (cfu/100 mL)	Absent	Absent	Absent	Absent	Absent
Salmonella spp. (cfu/100 mL)	Absent	Absent	Absent	Absent	Absent
Staphylococcus aureus (cfu/100 mL)	0	0	0	0	0
Molds and yeasts (cfu/100 mL)	<1	<1	<1	<1	<1
Total coliforms (cfu/100 mL)	0	0	0	0	0

Table S2; Correlation analysis between temperature and other physicochemical parameters that showed variation over time. The table shows correlation coefficients (r) and their corresponding significance p-values.

Parameter correlated with TEMPERATURE	Correlation Coefficient (r)	p-value
Free Residual Chlorine	-0.2495	$1.2244x10^{-04}$
Dissolved Aluminum	-0.5018	$3.3804x10^{-16}$
Total Alkalinity	-0.7294	$8.4149x10^{-40}$
Dissolved Calcium	0.1417	$3.0935x10^{-02}$
Dissolved Magnesium	0.4142	$4.9465x10^{-11}$
Sulphates	0.4114	$6.8373x10^{-11}$
pH	0.8414	$2.1137x10^{-63}$
Iron	0.7534	$9.3665x10^{-44}$
Trihalomethanes	0.9086	$3.5316x10^{-89}$

Figure S1: Relative abundance of all samples, water and biofilm, analysed at class level

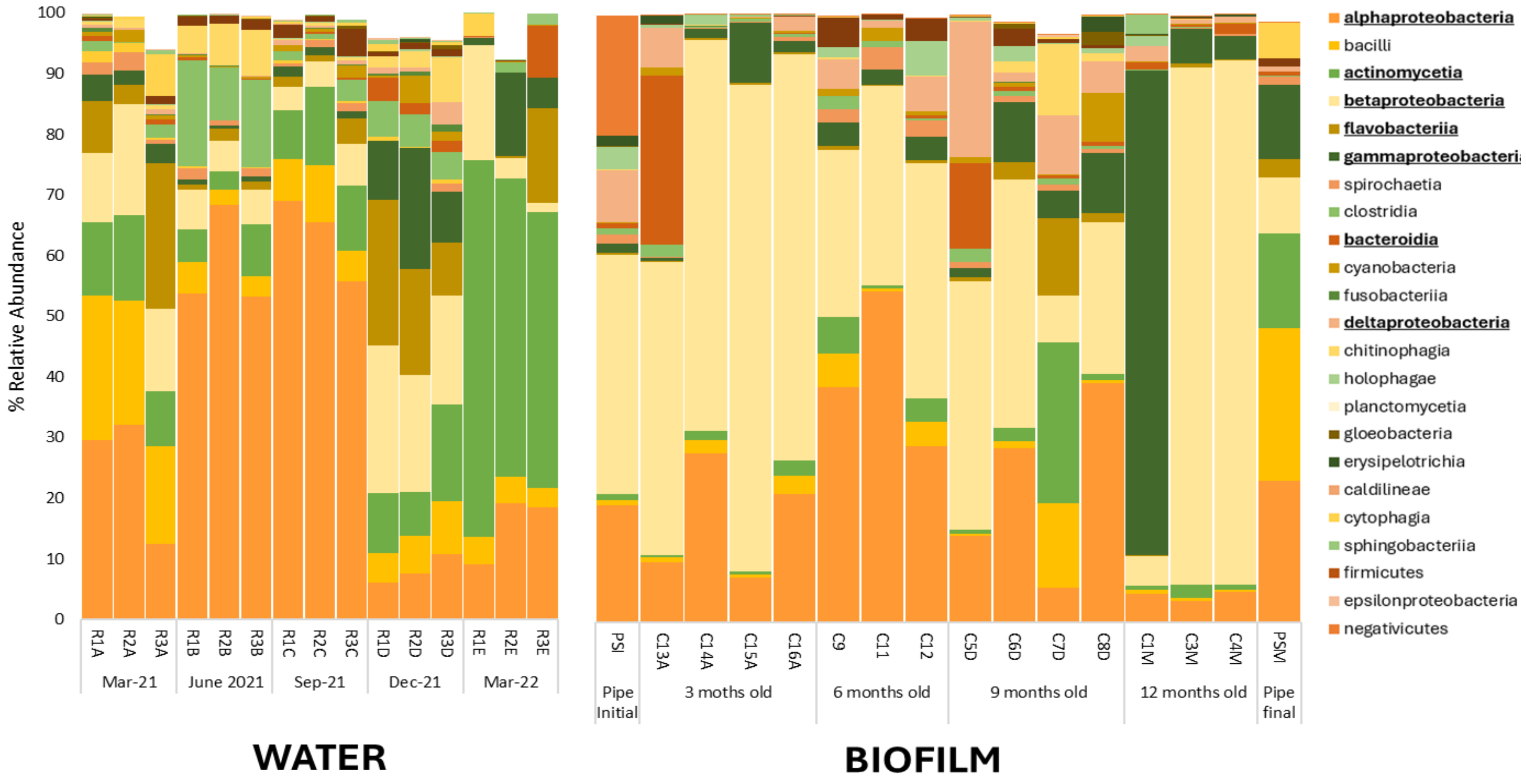

Figure S2: Bacterial relative abundance of all water samples at genera level (only most abundant genera are represented)

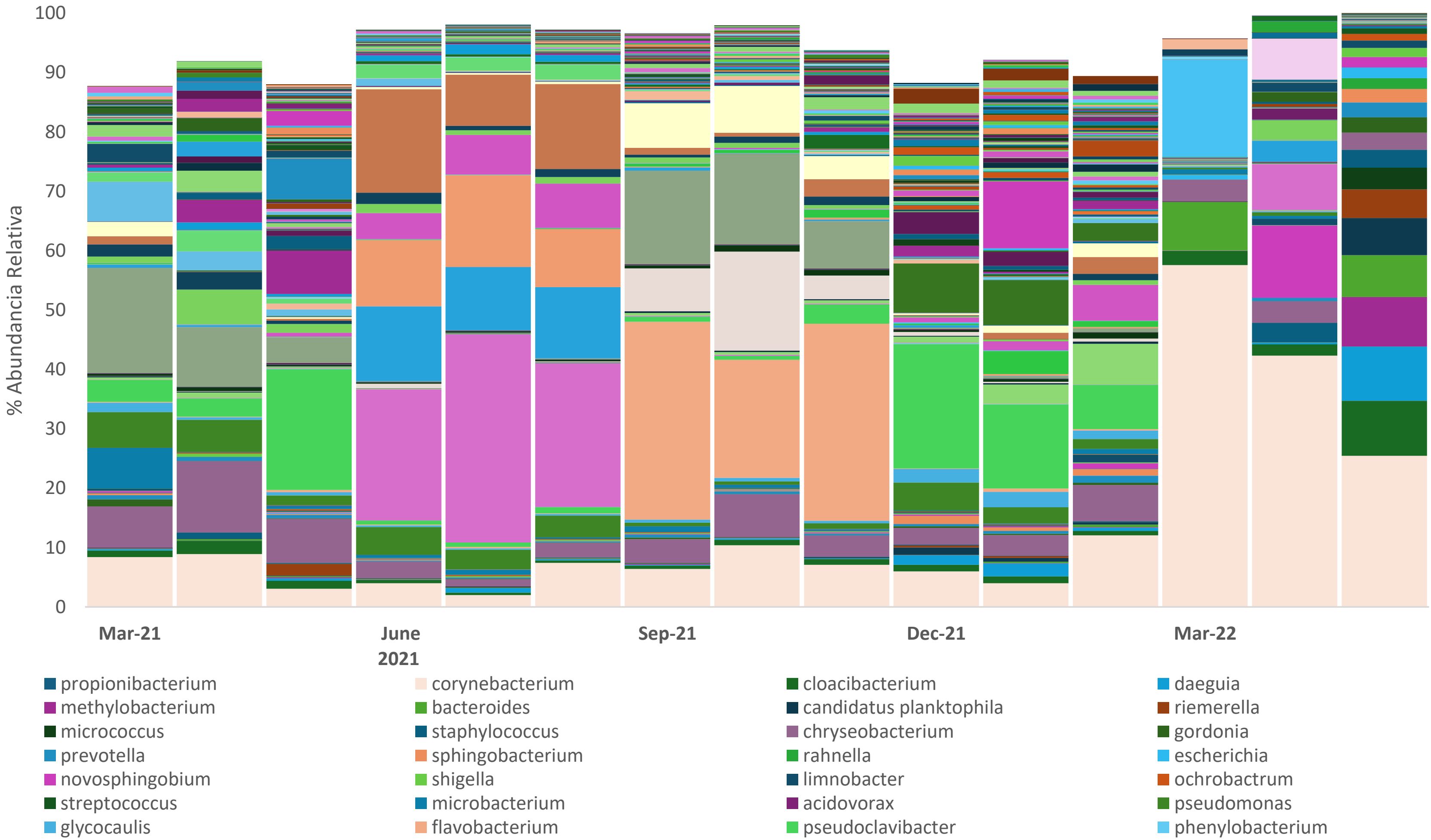

Figure S3: Bacterial relative abundance of all biofilm samples at genera level, only coupon used for succession studies

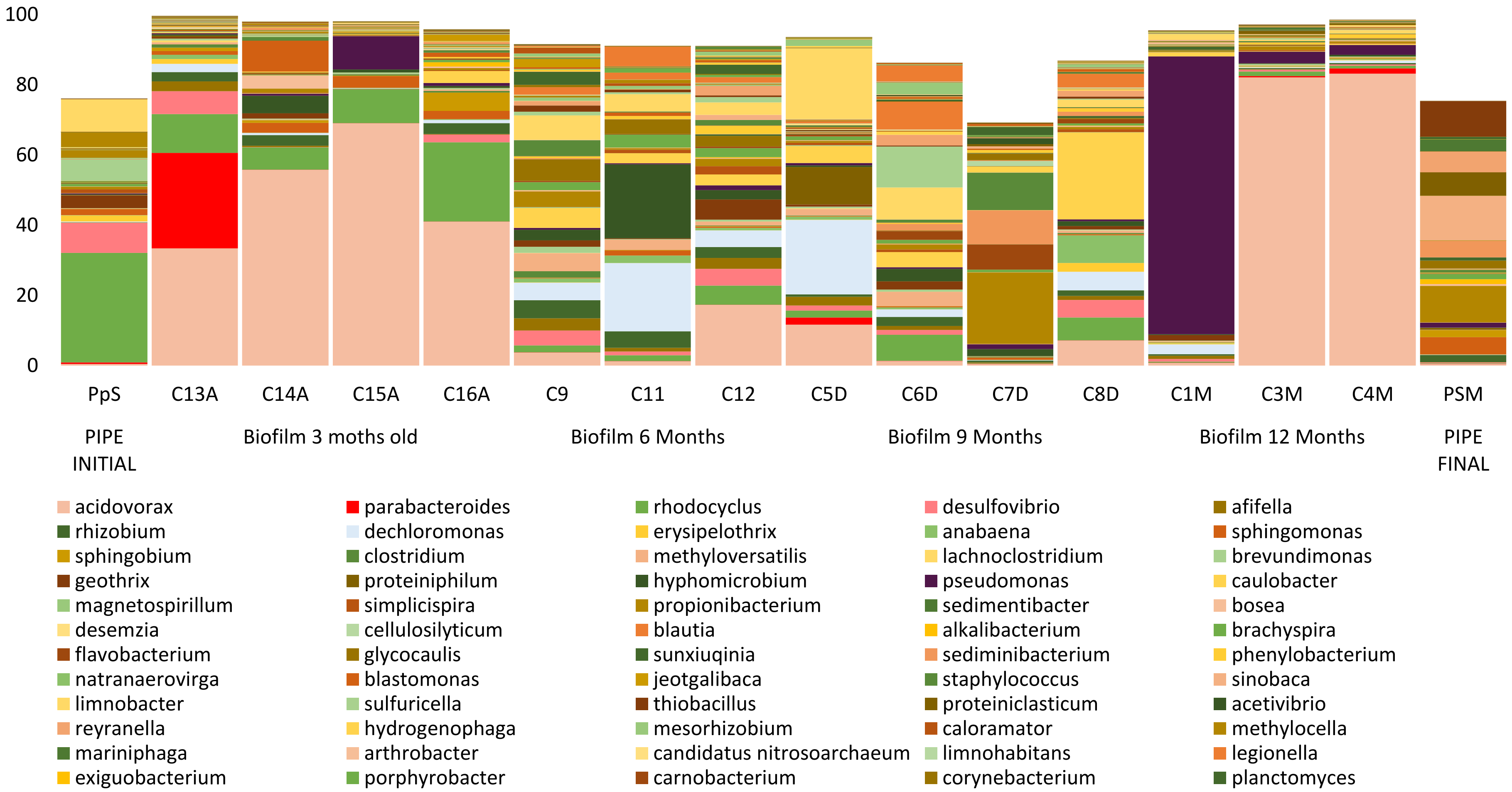

Supplement: Supplementary file 1 [file 11274_2026_5101_MOESM1_ESM.pdf]
